# The transmembrane protein LRIG2 increases tumor progression in skin carcinogenesis

**DOI:** 10.1002/1878-0261.12579

**Published:** 2019-10-21

**Authors:** Christine Hoesl, Thomas Fröhlich, Jennifer E. Hundt, Hermann Kneitz, Matthias Goebeler, Ronald Wolf, Marlon R. Schneider, Maik Dahlhoff

**Affiliations:** ^1^ Institute of Molecular Animal Breeding and Biotechnology Gene Center LMU München Germany; ^2^ Laboratory for Functional Genome Analysis (LAFUGA) Gene Center LMU München Germany; ^3^ Lübeck Institute for Experimental Dermatology Universität zu Lübeck Germany; ^4^ Klinik und Poliklinik für Dermatologie, Venerologie und Allergologie Universitätsklinikum Würzburg Germany; ^5^ Department of Dermatology und Allergology Philipps University Marburg Germany

**Keywords:** EGFR, ERBB receptors, LRIG2, mouse model, skin carcinogenesis, squamous cell carcinoma

## Abstract

Over the last few decades, the number of cases of non‐melanoma skin cancer (NMSC) has risen to over 3 million cases every year worldwide. Members of the ERBB receptor family are important regulators of skin development and homeostasis and, when dysregulated, contribute to skin pathogenesis. In this study, we investigated leucine‐rich repeats and immunoglobulin‐like domains 2 (LRIG2), a transmembrane protein involved in feedback loop regulation of the ERBB receptor family during NMSC. LRIG2 was identified to be up‐regulated in various types of squamous cell carcinoma (SCC), but little is known about LRIG2 in cutaneous SCC (cSCC). To investigate the function of LRIG2 in cSCC *in vivo*, we generated a skin‐specific LRIG2 overexpressing transgenic mouse line (LRIG2‐TG) using the Tet‐Off system. We employed the 7,12‐dimethylbenz(a)anthracene/12‐O‐tetra‐decanoylphorbol‐13‐acetate (DMBA/TPA) two‐stage chemical carcinogenesis model and analyzed the skin during homeostasis and tumorigenesis. LRIG2‐TG mice did not exhibit alterations in skin development or homeostasis but showed an interaction between LRIG2 and thrombospondin‐1, which is often involved in angiogenesis and tumorigenesis. However, during carcinogenesis, transgenic animals showed significantly increased tumor progression and a more rapid development of cSCC. This was accompanied by changes in the ERBB system. After a single TPA application, inflammation of the epidermis was enhanced during LRIG2 overexpression. In human skin samples, LRIG2 expression was identified in the basal layer of the epidermis and in hair follicles of normal skin, but also in cSCC samples. In conclusion, epidermal LRIG2 excess is associated with activated EGFR/ERBB4‐MAPK signaling and accelerated tumor progression in experimentally induced NMSC, suggesting LRIG2 as a potential oncoprotein in skin.

**Abbreviations**
AKT: RAC‐alpha serine/threonine‐protein kinase
BME: β‐mercaptoethanol
BrdU: bromodeoxyuridine
CASP3: caspase 3
CDH1: E‐cadherin
cSCC: cutaneous squamous cell carcinoma
EGF: epidermal growth factor
EGFR: EGF receptor
GAPDH: glyceraldehyde‐3‐phosphate dehydrogenase
HF: hair follicle
IB: immunoblotted
IL1A: interleukin‐1‐alpha
IL6: interleukin‐6
IP: immunoprecipitation
KRT: keratin
LOR: loricrin
LRIG: leucine‐rich repeats and immunoglobulin‐like domains
MAPK1/2: mitogen‐activated protein kinase 1/2
MKI67: proliferation marker protein Ki‐67
PCNA: proliferating cell nuclear antigen
PTEN: phosphatidylinositol 3,4,5‐triphosphate 3‐phosphatase and dual specificity protein phosphatase PTEN
RAS: GTPase Ras proteins
SHC1: SHC‐transforming protein 1
STAT: signal transducer and activator of transcription
THBS1: thrombospondin-1
TUBA1A: tubulin alpha‐1a chain
VIM: vimentin

## 1 Introduction

Excessive exposure to the sun and a history of sunburns are often linked to an increased incidence of malignant skin lesions (Kim and He, 2014; Rosso *et al.*, 1996). Every third cancer diagnosis is skin cancer, the most common type of cancer among Caucasians, with up to three million new non‐melanoma skin cancer (NMSC) cases per year worldwide (WHO, 2019). Increasing NMSC incidences demand the development of new therapies and prophylactic measures, as well as the optimization of screenings. NMSC arises from keratinocytes, and can be divided into basal cell carcinoma or cutaneous squamous cell carcinoma (cSCC) depending on the cell type from which tumors develop (Lapouge *et al.*, 2011; Sanchez‐Danes and Blanpain, 2018; Tan *et al.*, 2018). Dysregulated growth factors and their receptors have a deep impact on tumor initiation and progression (Witsch *et al.*, 2010). The epidermal growth factor receptor (EGFR, ERBB1, HER1) plays a crucial role in human cSCC (Rittie *et al.*, 2007). The EGFR and the other members of the ERBB receptor family (ERBB2‐4, HER2‐4) are widely expressed in human epidermis (Hoesl *et al.*, 2018) and regulate key processes of epidermal homeostasis, including proliferation, differentiation, and cell death. The deletion of ERBB4 in murine skin results in decreased epidermal thickness and keratinocyte proliferation (Hoesl *et al.*, 2018). Skin‐specific ERBB2 (Dahlhoff *et al.*, 2017) and ERBB3 (Dahlhoff *et al.*, 2015) knockout mice have been shown to play a major role of both receptors in NMSC promotion, and EGFR has been shown to play a crucial role in skin carcinogenesis (Chan *et al.*, 2004; Dahlhoff *et al.*, 2011; 2012). Signaling of the ERBB receptors is controlled by negative or positive feedback loops (Avraham and Yarden, 2011). During pathogenic processes, the dysregulation of those pathways can also influence ERBB signaling in a tumorigenic manner (Dahlhoff *et al.*, 2012). The leucine‐rich repeats and immunoglobulin‐like domains (LRIG) family comprises three transmembrane proteins (LRIG1‐3) (Guo *et al.*, 2004; Holmlund *et al.*, 2004; Nilsson *et al.*, 2001; Suzuki *et al.*, 1996) involved in the regulation of receptor tyrosine kinases (RTKs) such as the ERBB receptors (Guo *et al.*, 2004; Rafidi *et al.*, 2013). LRIG proteins have attracted attention especially due to their potential as prognostic markers in different cancer types (Lindquist *et al.*, 2014). In the skin, LRIG1 is predominantly expressed in a stem cell pool of the hair follicle (HF) (Jensen *et al.*, 2009), similarly to its expression in the intestine (Powell *et al.*, 2012) and stomach (Choi *et al.*, 2018), whereas LRIG2 and LRIG3 are expressed throughout the epidermis (Karlsson *et al.*, 2008). LRIG1 knockout mice develop psoriasis‐like skin lesions (Suzuki *et al.*, 2002). It has been shown that LRIG1 promotes EGFR, ERBB2, and ERBB3 degradation from the cell surface in a negative feedback loop (Gur *et al.*, 2004; Laederich *et al.*, 2004; Rubin *et al.*, 2005) and that the extracellular domain of LRIG1 decreases EGFR signaling in a paracrine manner (Yi *et al.*, 2011). LRIG3 opposes the function of LRIG1 and stabilizes the ERBB receptors at the cell surface of HEK293 cells (Rafidi *et al.*, 2013). Whereas tumor‐suppressive functions of LRIG1 (Mao *et al.*, 2018) and LRIG3 (Guo *et al.*, 2015) have been reported in malignant glioma, LRIG2 seems to act more as an oncoprotein (Holmlund *et al.*, 2009; Rondahl *et al.*, 2013; Xiao *et al.*, 2014). LRIG2 expression correlates with poor prognosis in SCC of the cervix and uterus, which show increased *LRIG2* RNA levels (Hedman *et al.*, 2010). It has also been shown that LRIG2 promotes EGFR signaling as a positive feedback loop in glioblastoma cells, supporting the hypothesis that LRIG2 is acting as an oncoprotein (Wang *et al.*, 2009; Xiao *et al.*, 2014; 2018). Importantly, although it is known that LRIG proteins can promote and suppress tumor growth in a tissue‐specific manner (Hedman and Henriksson, 2007), the molecular mechanisms and their impact on tumorigenesis in the skin are mostly unknown. The aim of this study was therefore to investigate the function of LRIG2 in the skin during development, homeostasis and tumorigenesis, and in particular its impact on the ERBB system. We consequently generated a skin‐specific transgenic (TG) mouse line overexpressing LRIG2 using the Tet‐Off system. LRIG2‐TG mice were viable and showed no major phenotype during development and homeostasis. When homeostasis was disrupted, the overexpression of LRIG2, however, resulted in increased inflammation, angiogenesis, tumor progression, and an early onset of cSCC, which affected the ERBB signaling and components of the extracellular matrix (ECM).

## 2 Materials and methods

### 2.1 Cell culture

HaCaT keratinocytes, A431, and A375 cells were purchased from CLS (Cell lines service, Eppelheim, Germany) no more than 4 months before the experiments were performed. All human permanent cell lines in the CLS cell bank have been authenticated using the STR DNA profiling analysis. Mycoplasma testing is done every 6 months for all cultured cells using a mycoplasma detection kit (PlasmoTest, InvivoGen, Toulouse, France). Cells were cultured in DMEM® medium (Biochrom, Berlin, Germany) and supplemented with 10% fetal calf serum (FCS; Biochrom), penicillin (100 U·mL^−1^), and streptomycin (100 µg·mL^−1^) (Biochrom) in a humidified incubator with 5% CO_2_ at 37 ˚C.

### 2.2 Human samples

Biopsy samples of cSCC were obtained from 10 patients ranging in age from 71 to 92 years, after written informed consent had been obtained. They were obtained at the Department of Dermatology, University Hospital Würzburg, Germany, and taken from the following anatomical sites: cheeks (3 patients), forehead (3 patients), nose, ear, dorsum of the hand and lower leg (1 patient each). Eight of these patients were diagnosed at stage I (pT1G1: 6 patients, pT1G2: 2 patients) and two at stage II (pT2G2 and pT2G3: one patient each) as classified according to the 8th Edition of the staging manual of the American Joint Committee on Cancer (AJCC‐8) (Califano *et al.*, 2017). Skin samples from non‐diseased skin of 10 individuals served as controls. Analysis of human tissue samples was approved by the Ethics Committee of the Medical Faculty, University of Würzburg, Germany (reference number #169/12) and the study methodologies conformed to the standards set by the Declaration of Helsinki.

### 2.3 Mice

Mice were maintained under specific‐pathogen‐free conditions and had access to water and standard rodent diet (V1534; Ssniff, Soest, Germany) *ad libitum*. C57BL/6N mice expressing the tetracycline‐regulated transcriptional transactivator (tTA) under the keratin 5 (KRT5) promoter have been originally described previously (Diamond *et al.*, 2000). We cloned murine *Lrig2* cDNA into the pTRE‐tight vector (Clontech, Mountain View, CA, USA) (pTRE‐tight‐LRIG2‐TG mouse line) or fused *Lrig2* cDNA with a sequence encoding the human influenza hemagglutinin (HA)‐epitope C‐terminally (pTRE‐tight‐HA‐LRIG2‐TG mouse line), and used these constructs to generate two independent TG mouse lines by pronuclear microinjection into zygotes of C57BL/6N mice. To obtain two independent TG KRT5‐LRIG2 mouse lines expressing transgenic LRIG2 skin‐specifically, the KRT5‐tTA mouse line was mated with either the pTRE‐tight‐LRIG2‐ or the pTRE‐tight‐HA‐LRIG2‐TG mouse line. Mouse strains were maintained in the C57BL/6N background. For further studies we used the HA‐tagged TG mouse line, referred to as LRIG2‐TG. To study proliferation rates of 12‐month‐old mice, 10 mm bromodeoxyuridine (BrdU; Roche, Mannheim, Germany) dissolved in PBS were injected intraperitoneal into the mice (30 mg·kg^−1 ^body weight) 3 hours before dissection.

To inhibit LRIG2‐TG expression, 3 mg·mL^−1^ doxycycline (Dox) [Beladox 500 mg·g^−1^, bela‐pharm (Lehnecke 793‐588), Schortens, Germany] and 5% sucrose (Sigma, Taufkirchen, Germany) was added to the drinking water for 2 weeks.

LRIG2‐TG mice and controls (Co) were dissected at indicated time points, skin samples were fixed in 4% paraformaldehyde (PFA; Sigma), dehydrated, and embedded in paraffin or snap‐frozen and stored at −80 °C until use. All murine experiments were approved by the Committee on Animal Health and Care of the local governmental body of the state of Upper Bavaria (Regierung von Oberbayern), Germany, and were performed in strict compliance with the European Communities Council Directive (86/609/EEC) recommendations for the care and use of laboratory animals.

### 2.4 Chemical skin carcinogenesis and TPA‐induced epidermal dysplasia

Chemical carcinogenesis was carried out according to internationally accepted standards as described elsewhere (Abel *et al.*, 2009). For tumor initiation, the carcinogen 7,12‐dimethylbenz(a)anthracene (100 µL DMBA dissolved in acetone, 400 nM; Sigma) was applied once to the shaved back skin of 7‐week‐old female LRIG2‐TG mice and controls. Tumor promotion was achieved by repeated application of the tumor promoting agent 12‐*O*‐tetra‐decanoylphorbol‐13‐acetate (50 µL TPA dissolved in ethanol, 10 nM; Sigma) twice a week for 24 weeks. Tumor development was assessed weekly.

To investigate the effect of LRIG2 during early hyperproliferative stages, shaved back skin of 9‐week‐old LRIG2‐TG mice and controls were exposed to a single dose of TPA (50 µL TPA dissolved in ethanol, 10 nM; Sigma). Mice were euthanized 48 h after TPA application. Skin samples were processed as described above.

### 2.5 Co‐immunoprecipitation and Western blot analysis

Protein was extracted by using Laemmli extraction buffer for skin samples or protein lysis buffer (0.05 m Hepes pH 7.5, 10% glycerol, 0.15 m NaCl, 1% Triton X‐100, 0.5 m EDTA, 0.5 m EGTA, 0.01 m NaF, 0.025 m β‐glycerol phosphate, 0.01 m Na_3_Vo_4_, Phosphatase inhibitor cocktail; Roche) for cell lysates or skin samples used for co‐immunoprecipitation (IP) experiments. Protein concentration was estimated by bicinchoninic acid protein assay. About 300 µg of total protein were used for co‐IP with 1.8 µg HA‐Tag antibody and Dynabeads^®^ Protein G (Invitrogen, Carlsbad, CA, USA). Protein lysates were pre‐cleared with Dynabeads^®^ Protein G for 60 min at 4 °C and immunoprecipitated with the HA‐Tag antibody conjugated to the beads for 2 h at 4 °C. Samples were washed and elution was done with 2× Laemmli extraction buffer by heating at 95 °C for 5 min. For Western blot analysis, half of the co‐IP eluate or 5‐20 µg of total protein were separated by SDS/PAGE, transferred to PVDF membranes (Millipore, Schwalbach, Germany), and immunoblotted (IB) against antibodies as indicated. For reference proteins as well as the total proteins, to analyze the phosphorylated state, we stripped the membranes by incubating them with a stripping buffer [2% SDS, 62.5 mm Tris/HCl, pH 6.7 and 100 mm β‐mercaptoethanol (BME)] for 40 min at 70 °C. Afterwards, membranes were washed, blocked, and incubated with the second primary antibody. All primary and secondary antibodies and their dilutions are provided in Table S1. Densitometrical analysis was done using imagej (http://rsb.info.nih.gov/ij).

### 2.6 Histology, immunohistochemistry, and morphometric analysis

Skin samples were either embedded in paraffin or snap‐frozen on dry ice and embedded in Tissue‐Tek^®^ O.C.T.™ Compound (Sakura Finetek, Alphen aan den Rijn, the Netherlands). Giemsa or hematoxylin and eosin (H&E)‐staining, immunofluorescence, and immunohistochemistry were performed as described previously (Hoesl *et al.*, 2018). Giemsa and H&E‐stained sections were employed for histological analysis. Immunohistochemical staining was performed for the analysis of LRIG2 expression in human tissue samples and the detection of proliferating cells (MKI67 or BrdU positive). Briefly, sections were boiled in 10 mm sodium citrate buffer (pH 6.0) for antigen retrieval, and the endogenous peroxidase was blocked with 3% H_2_O_2_ for 15 min. Slides were blocked with 5% serum from the secondary antibody host and incubated overnight at 4 °C with indicated antibodies. After being washed in Tris‐buffered saline solution, the slides were incubated for 1 h with appropriate secondary biotin‐conjugated antibodies followed by 30 min incubation with streptavidin‐biotin complex (Vector Laboratories, Burlingame, CA, USA). ImmPACT^®^ AMEC Red or DAB Peroxidase (HRP) substrate (Vector Laboratories) were used as chromogen. Counterstaining was performed with hematoxylin. Immunofluorescence stainings were performed using the above protocol, but without blocking endogenous peroxidase and without incubation with the streptavidin‐biotin complex. Additionally, the M.O.M. Immunodetection Basic kit (Vector Laboratories) was applied to murine sections if primary antibodies were raised in mice. All primary and secondary antibodies and their dilution are listed in Table S1. For morphometric investigations, three different H&E‐ or Giemsa‐stained back skin sections were analyzed. Per animal, 60 pictures covering a total length of 39.2 mm of back skin epidermis were taken with a 200× magnification lens using a Leica DFC425C digital camera (Leica Microsystems, Wetzlar, Germany). The area of all visible SGs was recorded with las software version 3.8.0 (Leica Microsystems) and employed to calculate the mean gland area. Epidermal thickness was investigated on the same sections on three constantly distributed measuring points per picture, resulting in a total of 180 measuring points per animal. To analyze the epidermal proliferation rate, BrdU‐ or MKI67‐stained sections were evaluated and the total number of epidermal nuclei and the total number of BrdU or MKI67 positive nuclei were similarly determined on 60 images covering a length of 39.2 mm.

### 2.7 Gelatin zymography

Gelatin zymography was performed as described previously (Reiter *et al.*, 2015). Briefly, protein samples (50 µg) lysed in protein lysis buffer were separated on an 8% acrylamide gel with 1% gelatin. Gels were incubated in a renaturation‐buffer (2.5% Triton X‐100 in H_2_O), followed by a 20 h developing step in the incubation buffer (500 mm TRIS, 2 m NaCl, 50 mm CaCl_2_, 50 µm ZnCl_2_) at 37 °C, stained with Coomassie Brilliant Blue R, and washed with decolorizing solution (5% methanol, 7% acetic acid). Proteinase activities were determined by densitometrical analysis of the inverse band intensities using imagej.

### 2.8 Mass spectrometry analysis

For mass spectrometry analysis reduced (8% BME) and non‐reduced protein samples of LRIG2‐TG back skin and controls were separated by SDS/PAGE. Gels were stained with Coomassie Brilliant Blue R, and protein bands above 300 kDa were excised. To reduce disulfide bonds, the gel slices were incubated in 45 mm dithioerythritol/50 mm NH_4_HCO_3_ for 30 min at 55 °C. Free sulfhydryl groups were blocked using 0.1 m iodoacetamide in 50 mm NH_4_HCO_3_ at room temperature for 2 × 15 min. For digestion, gel pieces were minced and covered with 100 ng porcine trypsin in 50 mm NH_4_HCO_3_ (Promega, Madison, WI, USA). Peptides were separated on a C18 column (PepMap RSLC, C18, 2 µm, 100A, 75 µm × 50 cm; Thermo Scientific, Rockford, IL, USA) at a flow rate of 200 nL·min^−1^ using an EASY‐nLC 1000 system (Thermo Scientific, Rockford, IL, USA). The gradients consisted of a 120 min ramp from 2% to 25% B (100% acetonitrile, 0.1% formic acid) and a consecutive ramp to 50% B within 10 min. Mass spectra were acquired using a top 5 data‐dependent method on an online coupled LTQ Orbitrap XL instrument (Thermo Scientific, Rockford, IL, USA). Spectra were searched using mascot V2.4 (Matrix Science Ltd, London, UK) and the murine subset of the UniProt database. For evaluation of the data, scaffold V 4.1 (Proteome Software, Inc, Portland, OR, USA) was used.

### 2.9 RNA expression analysis

Organs were homogenized in TRIzol reagent (Invitrogen, Darmstadt, Germany) for RNA isolation. 3 µg RNA were reverse‐transcribed in a final volume of 30 µL using RevertAid Reverse Transcriptase (Thermo Scientific, Schwerte, Germany) according to the manufacturer’s instructions. For qualitative analysis of mRNA expression of *HA‐Lrig2*, reverse transcription PCR (RT‐PCR) using reagents from Qiagen (Hilden, Germany) was performed. The final reaction volume was 20 μL, and cycle conditions were 94 °C for 5 min followed by 35 cycles of 94 °C for 1 min, 60 °C for 1 min, and 72 °C for 1 min. The following primers were employed: *HA‐Lrig2* forward primer 5′‐GAGGCAGGCAGCCATCAGC‐3′ and reverse primer 5′‐TCAAGCGTAGTCTGGGACG‐3′ and *Gapdh* forward primer 5′‐TCATCAACGGGAAGCCCATCAC‐3′ and reverse primer 5′‐AGACTCCACGACATACTCAGCACCG‐3′.

Quantitative mRNA expression analysis was performed by quantitative real‐time PCR (qRT‐PCR) using the StepOnePlus™ Real‐Time PCR System (Applied Biosystems, Waltham, MA, USA) and the PowerUp™ SYBR^®^ Green Master Mix (Applied Biosystems) according to the manufacturer’s instructions. The final primer concentration was 0.5 μm, and the final reaction volume was 20 μL, and cycle conditions were 95 °C for 2 min followed by 40 cycles of 95 °C for 15 s, 60 °C for 15 s, and 72 °C for 1 min. Quantitative values were obtained from the threshold cycle (*C*_T_) number, at which the increase in the signal associated with the exponential growth of PCR products begins to be detected. Transcribed RNA (cDNA) quantification was performed by using standard curves generated with a plasmid containing the murine *Lrig2* cDNA. We performed no‐template control and no‐RT control assays, which produced negligible signals with *C*_T_ values that were greater than 35. Experiments were performed in duplicates. The following primers were used: *Lrig2‐Fw*: 5′‐CACTGAAATACCTGAATTTGAGC‐3′, *Lrig2‐Rev*: 5′‐TCAGTTCCAAGAACTGGAGATG‐3′.

### 2.10 Statistical analysis

Data are presented as mean ± SEM and compared by Student’s *t*‐test (graphpad prism version 5.0 for Windows; GraphPad Software, San Diego, CA, USA), and in the case of more than two groups by ANOVA and Tukey’s multiple comparison test. Incidence, papilloma burden, and size were analyzed by 2‐way ANOVA. Group differences were considered to be statistically significant if *P* < 0.05.

## 3 Results

### 3.1 LRIG2 is expressed in human skin cancer

**Figure 1:**
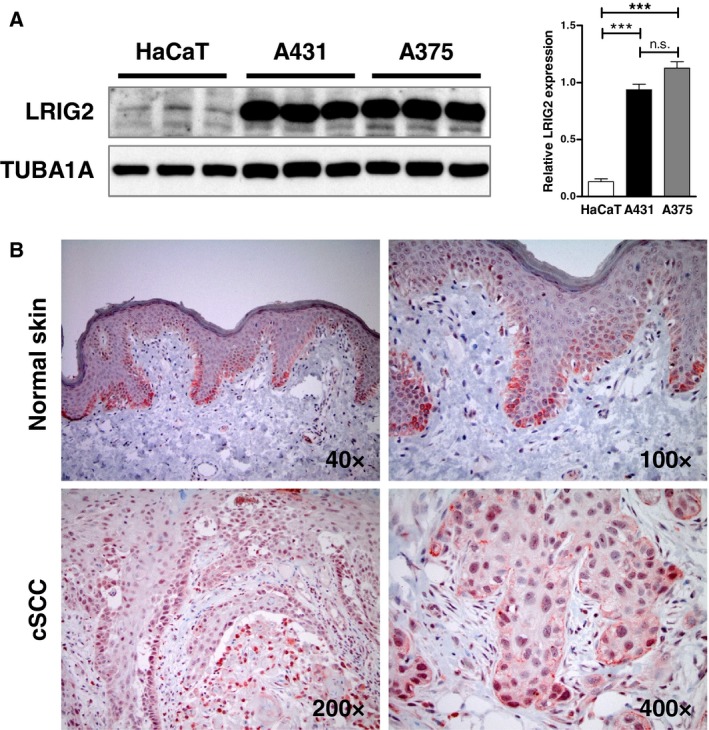
LRIG2 is expressed in human skin, cSCC, and human skin cell lines. (A) Western blot analysis of LRIG2 expression in HaCaT, A431, and A375 cells. TUBA1A was used as reference protein. Densitometric analysis of LRIG2 in relation to TUBA1A reveals that LRIG2 is significantly more highly expressed in both tumor cell lines compared with HaCaT keratinocytes. Data are presented as mean + SEM and were analyzed by ANOVA and Tukey’s multiple comparison test. ***P < 0.001. (B) Immunohistochemical visualization of LRIG2 expression (in red) in normal human skin and cSCC. Micrographs are representative for 10 cSCCs (8 patients with stage I and 2 patients with stage II according to AJCC‐8, Califano et al., 2017, see Materials and methods for details) and 10 normal skin samples. Magnification as indicated in the micrographs.

To evaluate the significance of LRIG2 in human skin homeostasis and tumorigenesis, we investigated LRIG2 expression in different human skin cell lines and tissue samples of healthy individuals and patients with cSCC. Western blot analysis revealed that LRIG2 expression was significantly increased in human cSCC (A431) and melanoma (A375) cell lines compared to human keratinocytes (HaCaT) (Fig. 1A). In normal human skin LRIG2 is predominantly expressed in the basal and lower spinous layer of the epidermis and in HFs with a mainly cytoplasmic pattern. In upper spinous layers, LRIG2 is also located in nuclei. All cSCC samples (10 cSCC samples/10 LRIG2 positive cSCC samples) revealed prominent LRIG2 expression in tumor cells with a predominantly nuclear staining pattern (Fig. 1B). These data indicate a role of LRIG2 during the pathogenesis of cSCC in humans.

### 3.2 Overexpression of LRIG2 has no influence on skin development and homeostasis

**Figure 2:**
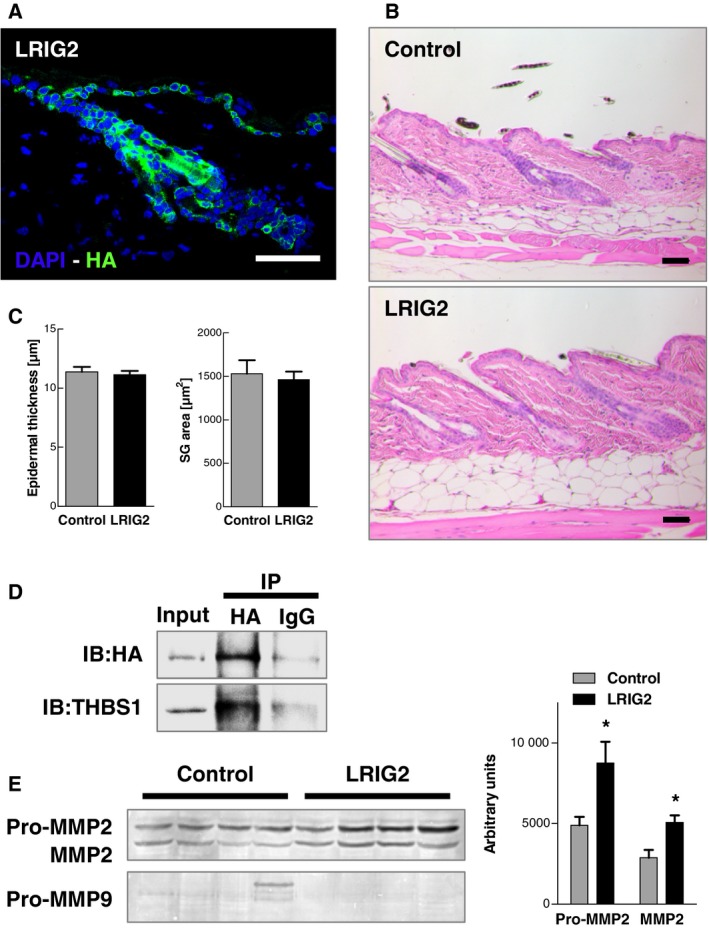
Skin‐specific overexpression of LRIG2 causes no phenotypical alterations. (A) Immunofluorescence staining against HA‐tag in the skin of a 12‐month‐old LRIG2‐TG animal demonstrates a strong expression of LRIG2 in epidermis, hair follicles, and sebaceous glands. HA‐tag in green and cell nuclei stained with DAPI (blue). Scale bar: 50 µm. (B) H&E staining of the skin of a 12‐month‐old LRIG2‐TG mouse and a control littermate. Scale bars: 50 µm. (C) Morphometric analysis of the epidermal thickness and sebaceous gland area revealed no alterations (*n* = 4). Data were analyzed by Student’s *t*‐test. (D) Co‐immunoprecipitation (IP) of HA‐tag in a LRIG2‐TG skin sample of a 12‐month‐old mouse. Immunoblotting (IB) revealed precipitation of LRIG2 and binding of THBS1. (E) Gelatin zymography of skin samples of 12‐month‐old LRIG2‐TG mice and controls (*n* = 4). Densitometric analysis of gelatin zymography revealed increased expression and activity of MMP2. Data are presented as mean + SEM and were analyzed by Student’s *t*‐test. *P < 0.01.

To investigate the function of LRIG2 in the skin, we generated two independent skin‐specific inducible transgenic mouse lines. We used the Tet‐Off system to induce the overexpression at a later time, in case a fetal overexpression turns out to be lethal. Both lines were mated with a keratin 5 promoter (KRT5‐tTA) driver mouse line. As both mouse lines showed no phenotype, in spite of an overexpression of *Lrig2* on RNA level (data not shown), the LRIG2 transgenic mouse line (LRIG2‐TG) with a C‐terminal HA‐tag was used for all experiments described in this manuscript. LRIG2‐TG mice were viable, showed no macroscopic phenotype, and bred in a Mendelian ratio (Fig. S1A). RT‐PCR (Fig. S1B), qRT‐PCR (Fig. S1D) and Western blot analysis (Fig. S1C) confirmed skin‐specific overexpression of the transgene. Western blots revealed that LRIG2‐TG animals treated for 2 weeks with doxycycline (Dox^+^) showed no transgene expression but endogenous LRIG2 levels comparable to those of control mice (Fig. S1E). LRIG2‐TG mice showed no altered expression of the other LRIG family members LRIG1 and LRIG3 (Fig. S1E). Immunofluorescence staining against the HA‐tag revealed expression of LRIG2 in the epidermis and HFs of transgenic animals (Fig. 2A). Histologically, LRIG2 overexpression had no effect on skin at any time under homeostatic conditions (Fig. 2B), not even in a long‐term study (up to 12 months). While the HF cycle was not impaired in LRIG2‐TG mice, they showed significantly more HFs in the late catagen phase VIII compared with controls on day P18 (Fig. S4). However, these changes seem to be transient, as such a finding could not be confirmed at any other time point. Epidermal thickness and sebaceous gland size showed no differences (Fig. 2C) between LRIG2‐TG animals and control littermates. In addition, epidermal differentiation and proliferation rate were unchanged in LRIG2‐TG mice (Fig. S2). Since LRIG proteins are feedback loop regulators of the ERBB receptor family, we analyzed ERBB expression and activation in the skin of LRIG2‐TG and control mice as well as their main target kinases mitogen‐activated protein kinase 1/2 (MAPK1/2) and RAC‐alpha serine/threonine‐protein kinase (AKT), but no differences became apparent (Fig. S3). Thus, we conclude that LRIG2 overexpression does not influence epidermal and HF development or homeostasis.

### 3.3 LRIG2 binds thrombospondin‐1

To identify potential interacting partners of the transmembrane protein LRIG2, we performed mass spectrometry analysis and co‐IPs. Investigation of LRIG2 expression in adult LRIG2‐TG and control mice under reducing and non‐reducing conditions by Western blot, revealed transgenic LRIG2 with a size of 120 kDa. Additionally, we detected a positive signal at 300 kDa, but only in non‐reduced LRIG2‐TG protein samples (Fig. S5A), which indicates the presence of proteins interacting with LRIG2. Corresponding bands of transgenic and control animals were analyzed by mass spectrometry to detect potential binding partners. Besides LRIG2, 40 further proteins were exclusively identified in transgenic animals and were sorted by total spectral counts of the non‐reduced LRIG2‐TG protein fraction. A table of the top 20 proteins is shown in Fig. S5B. We identified several keratins but also two glycoproteins, laminin subunit beta‐1 (LAMB1) and thrombospondin‐1 (THBS1), both containing EGF‐like motifs that possibly interact with LRIG2. In contrast to LAMB1, THBS1 has previously been shown to play a role in SCC and other cancers (Huang *et al.*, 2017). We therefore focused on THBS1 for further studies. THBS1 was exclusively identified in LRIG2‐TG samples by four individual peptides. Corresponding MS spectra and probability scores are shown in Fig. S5C,D. THBS1 has an important role in tyrosine kinase‐dependent signaling, is involved in angiogenesis and tumorigenesis, and mediates cell‐to‐cell and cell‐to‐matrix interactions. Immunoprecipitation revealed that THBS1 binds LRIG2, suggesting that THBS1 could be an important interaction partner of LRIG2 (Fig. 2D). While its expression was not increased in LRIG2‐TG animals, THBS1 may be stabilized by LRIG2 binding (Fig. S3). THBS1 regulates the matrix‐metalloproteinases (MMPs) 2 and 9 (Donnini *et al.*, 2004), which could be essential for tumor progression. We therefore analyzed MMP2 and MMP9 activity by zymography. LRIG2‐TG mice showed significantly increased levels of pro‐MMP2 and active MMP2, whereas no changes of MMP9 levels were detected (Fig. 2E). In summary, we identified THBS1 as a binding partner of LRIG2 and observed increased levels of pro‐ and active MMP2, an important modulator of the ECM, in the skin of LRIG2‐TG mice.

### 3.4 LRIG2 has a significant impact on progression of skin carcinogenesis

**Figure 3:**
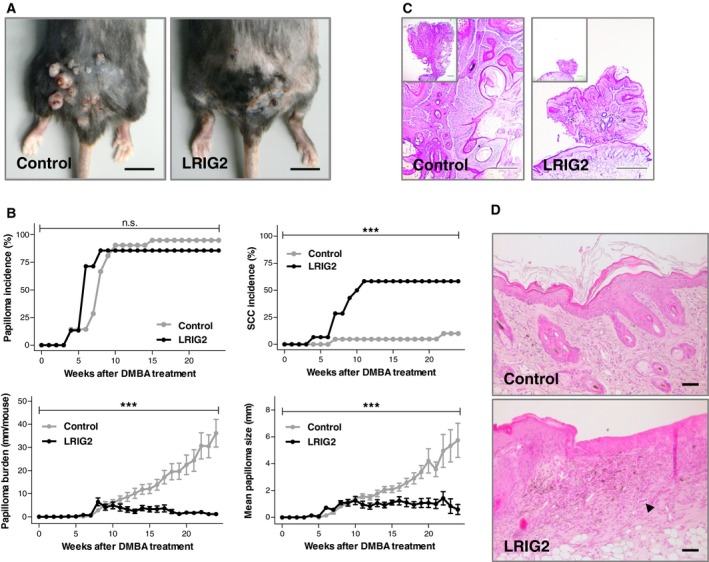
Accelerated development of cSCC in LRIG2‐TG mice in a chemically induced two‐step model of skin tumorigenesis. (A) Macroscopic pictures of the lower back skin of a representative LRIG2‐TG animal and a control littermate at the final stage. Scale bars: 1 cm. (B) Papilloma incidence, cSCC incidence, papilloma burden, and papilloma size of LRIG2‐TG animals compared with control littermates (*n* = 21 Controls/ 15 LRIG2‐TG). Data were analyzed by 2‐way ANOVA, and error bars represent SEM. Interaction: ***P < 0.001. n.s.: not significant. (C) H&E staining of a papilloma of a LRIG2‐TG mouse and a control littermate. Scale bars: 500 µm. (D) H&E staining of cSCC of a LRIG2‐TG mouse and back skin of a control littermate. Arrow points to tissue vascularization, indicating angiogenesis in LRIG2‐TG mice. Scale bars: 50 µm.

**Figure 4:**
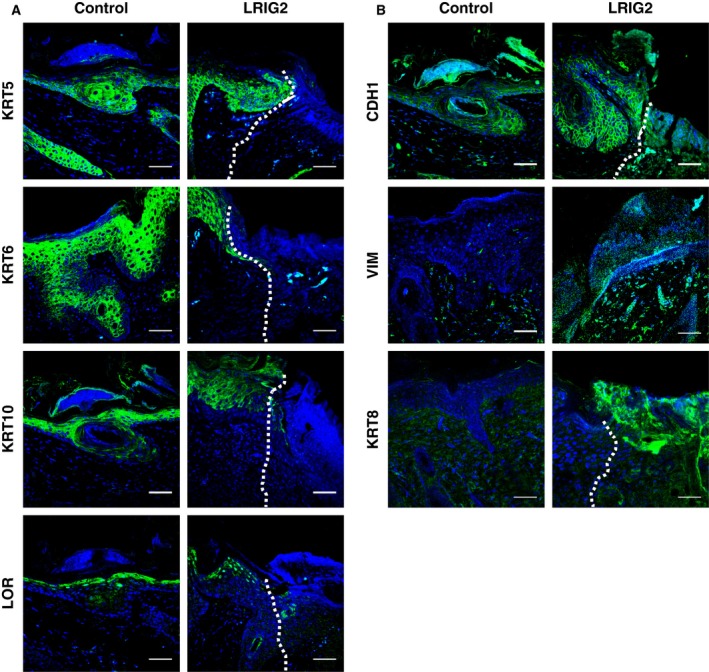
Epidermal differentiation during chemical induced skin tumorigenesis. (A) Immunofluorescence stainings against epidermal differentiation markers KRT5, KRT6, KRT10, and LOR (in green) (B) Immunofluorescence staining against CDH1, VIM, and KRT8 (in green). Cell nuclei are stained with DAPI (blue). Skin was obtained 24 weeks after initiation of chemically induced tumorigenesis. Shown are representative pictures of control skin including papillomata or close to papillomata and of LRIG2‐TG skin at the transition from epidermis to cSCC (white dashed line). Scale bars: 50 µm.

To determine whether LRIG2 affects skin tumorigenesis, we performed a two‐stage chemical skin carcinogenesis model with onetime application of DMBA on the back skin of LRIG2‐TG mice and control littermates causing tumor initiation followed by TPA treatment twice a week. In both groups, the first papillomata arose 4 weeks after DMBA treatment, without differences in tumor incidence, papilloma burden or size at this time. However, 10 weeks after tumor initiation we noticed a less pronounced increase of papilloma burden and papilloma size in LRIG2‐TG animals as compared with controls (Fig. 3B,C). Instead of papillomata, LRIG2‐TG mice developed a cSCC‐like phenotype on their backs, which started 6 weeks after tumor initiation (Fig. 3B). In all, 58% of the transgenic animals but only 10% of control littermates were affected (Fig. 3A,B). Histological and immunofluorescence analysis of the skin lesions revealed a phenotype resembling human cSCC. Atypical spindle‐like tumor cells arising from the epidermis protruded into the dermis and were accompanied by an inflammatory infiltrate (Fig. 3D). Moreover, vascularization appeared to be increased, which indicates angiogenesis (arrow, Fig. 3D). Tumors were stained for keratin 8 (KRT8), an established marker for cSCC in mice (Larcher *et al.*, 1992). The staining showed that KRT8 was highly expressed in the cSCC‐like lesions of LRIG2‐TG mice, but not in the controls (Fig. 4B). Additionally, cSCC‐like lesions of transgenic mice were poorly differentiated. In transgenic animals, the expression of epidermal differentiation markers [keratin 5 (KRT5), keratin 6 (KRT6), keratin 10 (KRT10), and loricrin (LOR), see Fig. 4A] in the cSCC‐like lesions decreased significantly in comparison with the adjacent epidermis, whereas the epidermis around the papillomata of controls was still differentiated. Moreover, epithelial polarity was lost. A decrease of epidermal E‐cadherin (CDH1) and a concomitant increase of vimentin (VIM) expression in the dermis indicate an enhanced tumor invasiveness, which might refer to epithelial–mesenchymal transition (EMT) (Fig. 4B). Altogether, our data suggest that LRIG2 has a tumor promoting function in murine skin, which results in an accelerated onset of cSCC development.

### 3.5 Increased EGFR and ERBB4 expression during tumor progression upon LRIG2 overexpression

**Figure 5:**
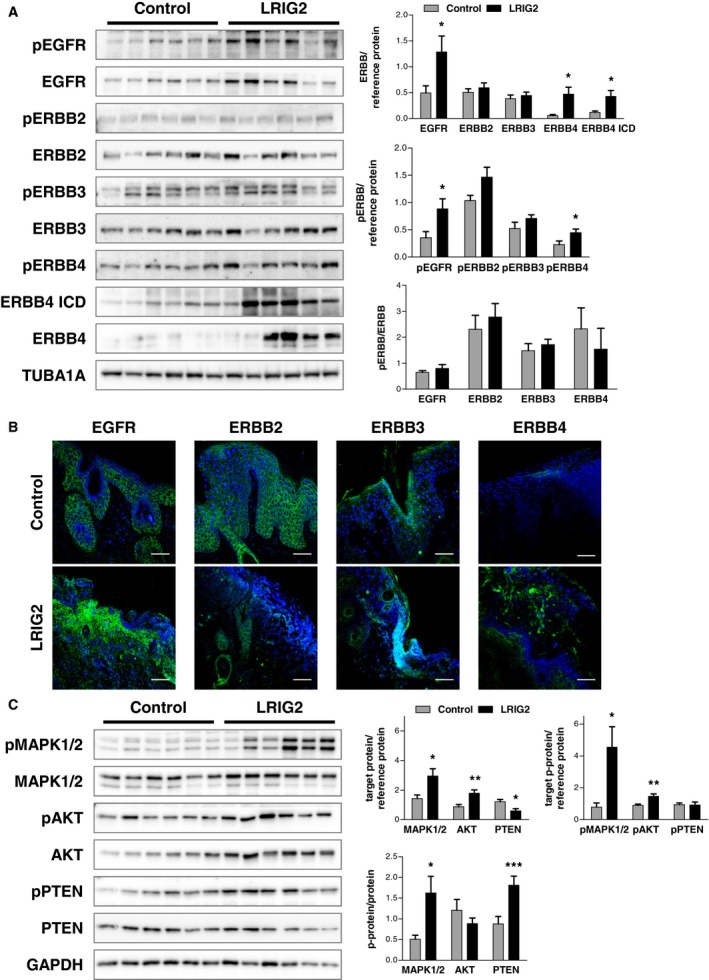
ERBB receptor expression during chemically induced skin tumorigenesis. (A) Western blot and densitometric analysis of phosphorylated ERBB receptors and ERBB receptors in skin samples obtained 24 weeks after initiation of chemically induced tumorigenesis. TUBA1A was used as reference protein (*n* = 6). (B) Immunofluorescence staining against ERBB1‐4 receptors (in green) using back skin sections from the carcinogenesis experiment of control and LRIG2‐TG mice. Scale bars: 50 µm. (C) Western blot and densitometric analysis of phosphorylated and total downstream targets of ERBB receptors (MAPK1/2, AKT, and PTEN). GAPDH was used as reference protein (*n* = 6). Data are presented as mean + SEM and were analyzed by Student’s *t*‐test. *P < 0.05, **P < 0.01, ***P < 0.001.

To investigate whether the tumor promoting activity of LRIG2 is ERBB receptor‐dependent, we analyzed the expression of the latter and respective downstream targets in transgenic and control skin during two‐stage skin carcinogenesis. Immunofluorescence and Western blot analyses revealed an increased expression of EGFR and ERBB4 in the cSCC‐like lesions of LRIG2‐TG mice (Fig. 5A,B). Concomitantly, the intracellular domain (ICD) of ERBB4 was significantly increased in LRIG2‐TG mice (Fig. 5A), which indicates that the receptor undergoes regulated intramembrane proteolysis. Thus the ICD can translocate to the nucleus and act as transcription factor. Additionally, phosphorylated ERBB4 and phospho‐EGFR were significantly increased in LRIG2‐TG mice compared with control littermates. We identified increased levels of AKT and phosphorylated AKT, a typical downstream target of the ERBB receptors, and phosphorylation of MAPK1/2 was significantly increased in transgenic animals compared with controls (Fig. 5C). The activity of other downstream targets such as SHC‐transforming protein 1 (SHC1), signal transducer and activator of transcription 3 (STAT3), STAT5, and GTPase Ras proteins (RAS) was unchanged (data not shown). The phosphorylation of phosphatidylinositol 3,4,5‐triphosphate 3‐phosphatase and dual specificity protein phosphatase PTEN (PTEN) was significantly increased, whereas total PTEN was decreased. This implies the loss of the tumor‐suppressive function of PTEN (Fig. 5C). Western blot analysis of THBS1 and zymography analysis for MMP2 and MMP9 revealed no differences between LRIG2‐TG and control animals (data not shown). In summary, these data indicate that during tumorigenesis LRIG2 increases skin tumor progression, associated with activation of EGFR/ERBB4‐MAPK signaling.

### 3.6 LRIG2 impairs TPA‐induced epidermal hyperplasia

**Figure 6:**
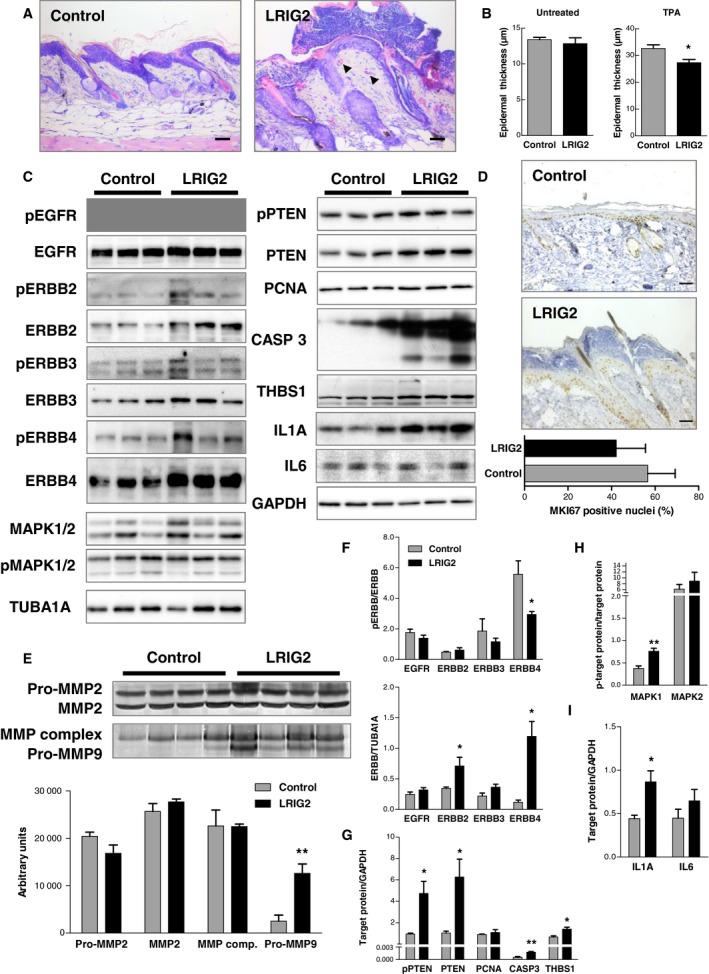
TPA induces an increased inflammation of the skin of LRIG2‐TG mice. (A) Giemsa staining reveals huge inflammation spots and blood vessels (arrows) in the skin of LRIG2‐TG mice 48 h after a single application of TPA. Scale bars: 50 µm. (B) Morphometric measurements of the epidermal thickness showed less pronounced increase of epidermal thickness in LRIG2‐TG mice compared with controls after TPA treatment (*n* = 4). (C) Western blot of phosphorylated and total ERBB receptors, MAPK1/2, PTEN, PCNA, CASP3, THBS1, and the inflammation markers: IL1A and IL6. GAPDH or TUBA1A were used as reference protein. (D) The proliferation index is not altered in LRIG2‐TG mice compared with controls (*n* = 4). MKI67 staining of back skin of a TPA‐treated TG and control mouse. Scale bars: 50 µm. (E) Gelatin zymography with densitometric analysis of skin samples of TPA‐treated LRIG2‐TG mice and controls (*n* = 4) revealed increased expression of pro‐MMP9. (F–I) Densitometric analysis of western blots in (C). Data are presented as mean + SEM and were analyzed by Student’s *t*‐test. *P < 0.05; **P < 0.01.

Our data suggest that LRIG2 is involved in tumor progression and accelerates tumorigenesis. To investigate an early point of time we induced epidermal hyperplasia by application of a single dose of TPA. In comparison with control mice, the increase of epidermal thickness upon TPA treatment was less pronounced in LRIG2‐TG animals (Fig. 6A,B). These, however, developed a more prominent neutrophil‐dominated inflammation (Fig. 6A). Western blot analysis revealed that the proinflammatory cytokine interleukin‐1‐alpha (IL1A) was significantly increased in LRIG2‐TG mice, whereas interleukin‐6 (IL6) was unchanged (Fig. 6C,I). As previously observed in our carcinogenesis model, ERBB4 was up‐regulated in the back skin of LRIG2‐TG mice 48 h after TPA treatment, but the fraction of phosphorylated ERBB4 was reduced. The other ERBB receptors were unchanged – with the exception of ERBB2, which exhibited higher expression in LRIG2‐TG mice compared with control littermates after TPA treatment (Fig. 6C,F). Additionally, we found increased activation of MAPK1 in LRIG2‐TG TPA‐treated skin, whereas MAPK2 was not affected (Fig. 6C,H). Importantly, PTEN expression levels were increased in LRIG2‐TG mice, but appeared to be phosphorylated and therefore inactivated (Fig. 6C,G). In accordance with a less prominent increase of epidermal thickness we found significantly increased levels of cleaved caspase‐3 (CASP3) in the skin of TPA‐treated LRIG2‐TG mice than in controls (Fig. 6C,G), whereas the proliferation rate was unchanged (Fig. 6C,D,G). Moreover, LRIG2‐TG mice revealed a significant increase in THBS1 expression (Fig. 6C,G) and a significant up‐regulation of pro‐MMP9 (Fig. 6E). Unlike the findings under homeostatic conditions, however, MMP2 activity was not affected by LRIG2 overexpression due to TPA treatment (Fig. 6E). In summary, LRIG2 overexpression leads to an increased inflammatory response after TPA treatment, which might contribute to tumorigenesis.

## 4 Discussion

LRIG proteins are important regulators of different RTKs and are involved in negative and positive feedback loops of the ERBB receptor family (Avraham and Yarden, 2011). LRIG1 and LRIG3 show mostly tumor‐suppressive function, whereas LRIG2 frequently seems to act as an oncoprotein (Lindquist *et al.*, 2014). Increased LRIG2 expression correlates with a poorer prognosis in patients with oligodendroglioma (Holmlund *et al.*, 2009), cervical SCC (Hedman *et al.*, 2010), non‐small cell lung cancer (Wang *et al.*, 2014), and glioblastoma (Wang *et al.*, 2009). Ubiquitous LRIG2 knockout mice were protected against glioblastoma, demonstrating that LRIG2 plays a crucial role in glioblastoma initiation and progression (Rondahl *et al.*, 2013). It has been shown that the extracellular domain of the transmembrane LRIG2 protein is the part of the protein that is required to mediate the proliferative effect during glioblastoma progression (Xiao *et al.*, 2014). This is an important finding, as the extracellular domain of a protein is usually a more amenable drug target. Nevertheless, tumorigenic activity of LRIG proteins is often tissue‐specific (Hedman *et al.*, 2010; Holmlund *et al.*, 2009; Lindquist *et al.*, 2014) and nothing is known about the function of LRIG2 in the skin and skin tumorigenesis. We identified LRIG2 expression in a human keratinocyte cell line (HaCaT), in an epidermal tumor cell line (A431), in a human melanoma cell line (A375), and in human tissue samples of cSCC patients and normal skin. Our study revealed increased LRIG2 expression in cancer cells *in vitro*, indicating a tumorigenic function of LRIG2. Interestingly, LRIG2 expression is mostly cytoplasmic in normal, basal epidermis, but nuclear in more differentiated epidermal layers and in cancer cells. LRIG proteins show an altered localization in psoriasis (Karlsson *et al.*, 2008). The nuclear localization of LRIG2 in our cSCC tissue samples may also indicate a proliferative and pathogenic function.

To investigate the impact of LRIG2 during skin development, homeostasis, and tumorigenesis *in vivo*, we generated a skin‐specific LRIG2‐TG mouse model. Long‐term studies of the mouse line revealed no major phenotypical changes under homeostatic conditions. LRIG2 had no impact on epidermal thickness, sebaceous gland size, epidermal differentiation or proliferation. By employing proteomic analysis, we identified THBS1 as a potential binding partner of LRIG2. THBS1 attracted our attention because of its EGF‐like motifs (Carlson *et al.*, 2008), its function in the modulation of the ECM, angiogenesis, and its implication in SCC and other types of cancer (Donnini *et al.*, 2004; Huang *et al.*, 2017; Qian *et al.*, 1997; Tan and Lawler, 2009). The binding of THBS1 could be related to an increase of pro‐MMP2 and active MMP2, which is essential for tumor cell invasion, inflammation, and neovascularization (Hernandez‐Perez *et al.*, 2012). Although these effects of LRIG2 have no obvious impact on skin homeostasis, LRIG2‐TG mice showed an increased tumor progression compared with control littermates during two‐stage chemical skin carcinogenesis. Animals showed no differences in tumor initiation, but at the end of the experiment 58% of all LRIG2‐TG mice, compared with only 10% of all controls, developed skin tumors resembling human cSCC. LRIG2‐TG mice showed downward invasion of atypical cells, neovascularization, and inflammation, accompanied by KRT8 expression and the loss of epidermal differentiation markers. Additionally, the decreased expression of CDH1 and concomitant increase of VIM suggest an EMT (Kang and Massague, 2004; Navarro *et al.*, 1991) in LRIG2‐TG animals, a process essential for cell–cell interaction and cSCC progression. Analysis of ERBB receptor expression revealed an increase of EGFR and ERBB4 and their downstream targets MAPK and AKT. The autonomous EGFR and ERBB4 receptors are important for tissue development and homeostasis, but they also play a major role in tumorigenesis, particularly in skin cancer (Citri and Yarden, 2006; Holbro and Hynes, 2004; Yarden and Sliwkowski, 2001). Additionally, we found the ICD of ERBB4 increased in cSCC tissue samples of LRIG2‐TG mice, which may act as a transcription factor and have an important impact on tumorigenesis (Haskins *et al.*, 2014; Maatta *et al.*, 2006). Increased levels of phosphorylated MAPK and AKT and the concomitant loss of PTEN phosphatase activity are often observed in rapid and aggressive tumorigenesis (Segrelles *et al.*, 2002; Yang *et al.*, 2015). LRIG2 overexpression seems to influence tumor suppressor PTEN activity, which may explain the dramatic phenotype during skin carcinogenesis and the absence of effects in skin homeostasis. Phosphorylation and thus inactivation of PTEN at residues Ser380/Thr382/383 is significantly increased in LRIG2‐TG mice 24 weeks after initiation of chemically induced tumorigenesis (Yang *et al.*, 2015). As the altered molecular signaling may reflect the differences between papillomata and cSCC tissue, we additionally analyzed tumor initiation in the transgenic LRIG2 model. To investigate the very early tumor initiation in more detail, we performed an epidermal hyperplasia experiment with a single TPA application. LRIG2‐TG mice showed increased inflammatory cell infiltration and neovascularization, which can be an indication for tumor promotion and progression. These findings were additionally confirmed by an increase of IL1A expression (Salven *et al.*, 2002) and the up‐regulation of ERBB2 and ERBB4 in the skin of LRIG2‐TG mice. EGFR, however, was not increasingly expressed or phosphorylated at this early stage, but the increased expression of PTEN seems to be phosphorylated and thus inactivated. Inactivation of PTEN plays an important role in human cSCC development (Darido *et al.*, 2018; Hertzler‐Schaefer *et al.*, 2014), consequently the loss of PTEN tumor‐suppressive function might be an important element of LRIG2‐mediated tumor progression during skin cancer. Furthermore, it was shown that THBS1 up‐regulates MMP9 expression in endothelial cells and promotes tumor cell invasion (Qian *et al.*, 1997). We found the expression of THBS1 and MMP9 to be increased in LRIG2‐TG mice 48 h after TPA application, assuming that THBS1 expression could be involved in LRIG2‐mediated tumor initiation and progression (Tan and Lawler, 2009). THBS1 may induce angiogenesis in tumors, influence tumor cell adhesion, migration, invasion, and metastasis both *in vitro* and *in vivo* (Tuszynski and Nicosia, 1996). Additionally, THBS1 is expressed in tumor cells, showing tumor progressive function. THBS1 also induces auto‐phosphorylation of EGFR in A431 cells (Liu *et al.*, 2009). The above supports a relationship with the ERBB receptor system. The increase of inflammatory cells such as macrophages and monocytes, however, may also have led to the increase of THBS1 (Lopez‐Dee *et al.*, 2011). Our findings during early hyperproliferative stages point to a tumor‐promoting influence of LRIG2 excess. In contrast, we found no alterations in THBS1 or MMP levels during skin carcinogenesis. The up‐regulation of pro‐MMP9 during TPA approach and the increase of pro‐ and active MMP2 may be overlaid by a general increase of MMP2 and MMP9 in cSCC as previously described (Dumas *et al.*, 1999).

## 5 Conclusions

In conclusion, our study reveals an important function of LRIG2 during skin carcinogenesis. In human skin, LRIG2 is expressed in the HF and in the basal layer of the epidermis, and our preliminary data indicate that its expression is increased in skin cancer cell lines. Furthermore, we detected LRIG2‐positive cells as a frequent feature of human cSCC samples. Even though LRIG2 overexpression has no obvious major impact on skin development and homeostasis, LRIG2 may promote tumor growth and induce a more severe carcinogenic phenotype, possibly by inactivating the tumor suppressor PTEN. Our results show an early onset of cSCC in LRIG2‐TG mice during two‐stage chemical skin carcinogenesis accompanied by altered ERBB signaling.

## Conflict of interest

The authors declare no conflict of interest.

## Author contributions

Conception and design: M.D., C.H., M.R.S. Development of methodology: M.D., C.H., M.R.S. Acquisition of data: M.D., R.W., M.G., J.E.H. Analysis and interpretation of data: M.D., C.H., H.K., T.F., J.E.H. Writing, review, and/or revision of the manuscript: M.D., C.H., M.R.S. Study supervision: M.D. Discussion of the experiments at planning stage and discussions of the results: M.D., C.H., M.R.S., M.G., H.K., T.F., J.E.H.

## Supporting information


**Fig. S1.** LRIG2 expression.

**Fig. S2.** Epidermal differentiation and proliferation analysis.

**Fig. S3.** Western blot analysis of the ERBB receptors and their downstream targets.

**Fig. S4.** Hair cycle analysis.

**Fig. S5.** Mass spectrometry data.

**Table S1.** Antibodies employed for Western blots analysis, immunoprecipitation, immunohistochemistry, and immunofluorescence.
