# Supplementary material for: The transmembrane protein LRIG2 increases tumor progression in skin carcinogenesis
Source: Mol Oncol. 2019 Oct 21;13(11):2476–92. doi: 10.1002/1878-0261.12579 (PMC6822252; doi:10.1002/1878-0261.12579)
Supplement: Supplementary file 1 — Fig. S1. LRIG2 expression. [file MOL2-13-2476-s001.pdf]

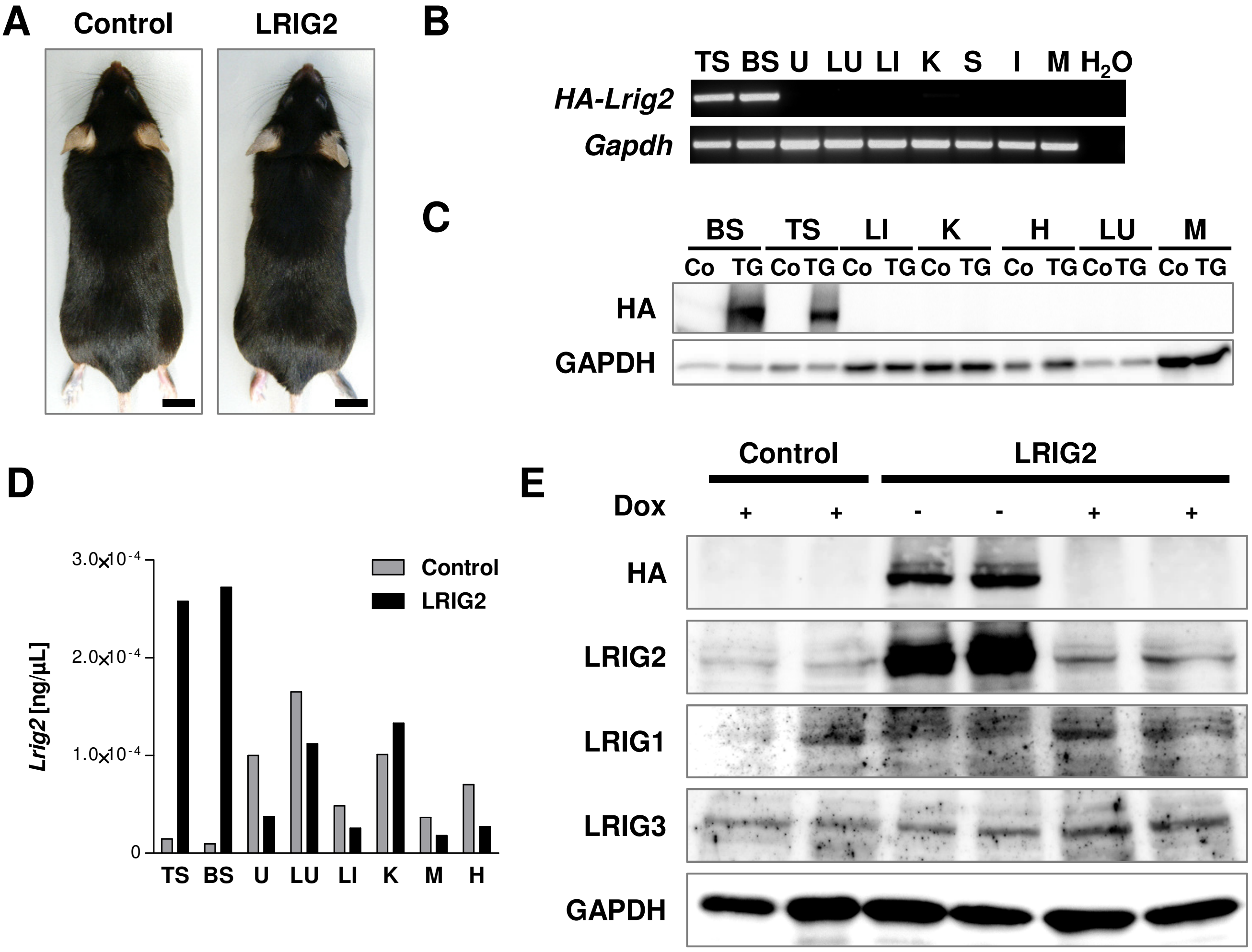

Figure S1. **(A)** Macroscopic appearance of a representative LRIG2-TG animal and a control littermate. Scale bars represent 1 cm. **(B)** Reverse transcription PCR shows transgene expression only in tail and back skin. *Gapdh* is used as reference gene. **(C)** Western blot analysis revealed the expression of a HA-tag only in the skin of TG mice. GAPDH was used as reference protein. **(D)** Quantification of *Lrig2* mRNA in indicated organs by quantitative real-time PCR. **(E)** Western blot analysis of Ha-tag and LRIG proteins of the back skin of LRIG2-TG mice and control littermates. To inhibit LRIG2-TG overexpression doxycycline (3 mg/mL) was added to the drinking water for two weeks. GAPDH was used as reference protein. All mice were female and six months old when they were dissected. TS: tail skin, BS: back skin, U: uterus, LU: Lung, LI: liver, K: kidney, S: spleen, I: intestine, M: muscle, H: heart, Dox: doxycycline, +: mice received 3 mg/ml Dox in the drinking water for two weeks, -: mice received no Dox.

Hoesl et al.
Fig. S1
