# Supplementary material for: The transmembrane protein LRIG2 increases tumor progression in skin carcinogenesis
Source: Mol Oncol. 2019 Oct 21;13(11):2476–92. doi: 10.1002/1878-0261.12579 (PMC6822252; doi:10.1002/1878-0261.12579)
Supplement: Supplementary file 2 — Fig. S2. Epidermal differentiation and proliferation analysis. [file MOL2-13-2476-s002.pdf]

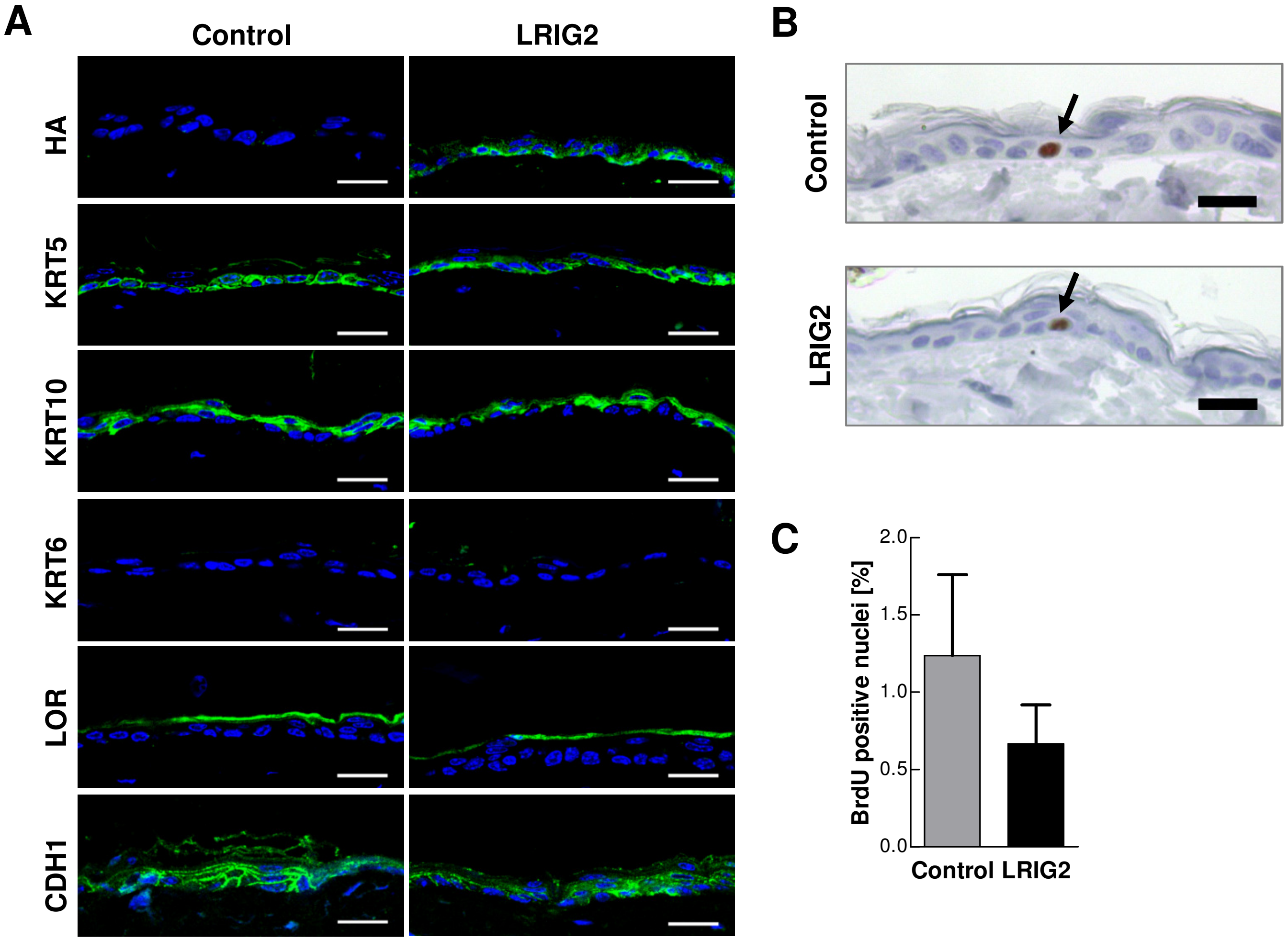

Figure S2. (A) Immunofluorescence staining against the differentiation markers KRT5, KRT10, KRT6, LOR, CDH1 and the HA-tag (all in green) using back skin sections of six-month-old control and LRIG2-TG mice. Cell nuclei are stained with DAPI (blue). **(B)** BrdU staining of back skin of a twelve-month-old TG and control mouse. Arrows indicate BrdU positive, proliferating cells. **(C)** Proliferation index is not altered in LRIG2-TG mice compared to controls. Data are presented as mean+SD and were analyzed by Student's *t*-test. Scale bars in **(A)** and **(B)** represent 20 µm.

Hoesl et al.
Fig. S2
