# Supplementary material for: The transmembrane protein LRIG2 increases tumor progression in skin carcinogenesis
Source: Mol Oncol. 2019 Oct 21;13(11):2476–92. doi: 10.1002/1878-0261.12579 (PMC6822252; doi:10.1002/1878-0261.12579)
Supplement: Supplementary file 3 — Fig. S3. Western blot analysis of the ERBB receptors and their downstream targets. [file MOL2-13-2476-s003.pdf]

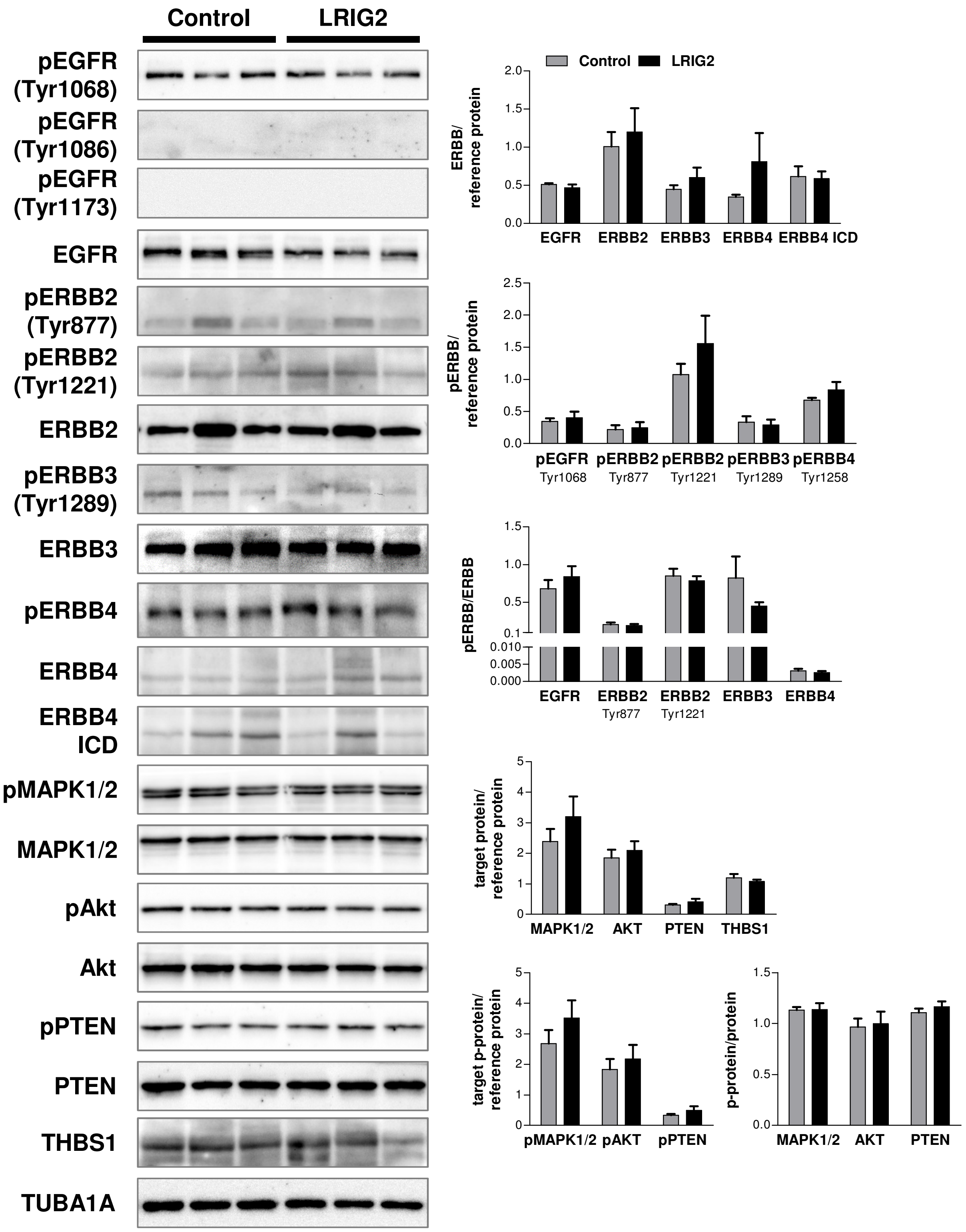

Figure S3. Western blot and densitometrical analysis of phosphorylated and non-phosphorylated ERBB receptors, MAPK1/2, AKT and PTEN and THBS1 of the back skin of twelve-month-old LRIG2-TG mice and control littermates. TUBA1A was used as reference protein. Data are presented as mean+SEM and were analyzed by Student's *t*-test.

Hoesl et al.
Fig. S3
