# Supplementary material for: The transmembrane protein LRIG2 increases tumor progression in skin carcinogenesis
Source: Mol Oncol. 2019 Oct 21;13(11):2476–92. doi: 10.1002/1878-0261.12579 (PMC6822252; doi:10.1002/1878-0261.12579)
Supplement: Supplementary file 4 — Fig. S4. Hair cycle analysis. [file MOL2-13-2476-s004.pdf]

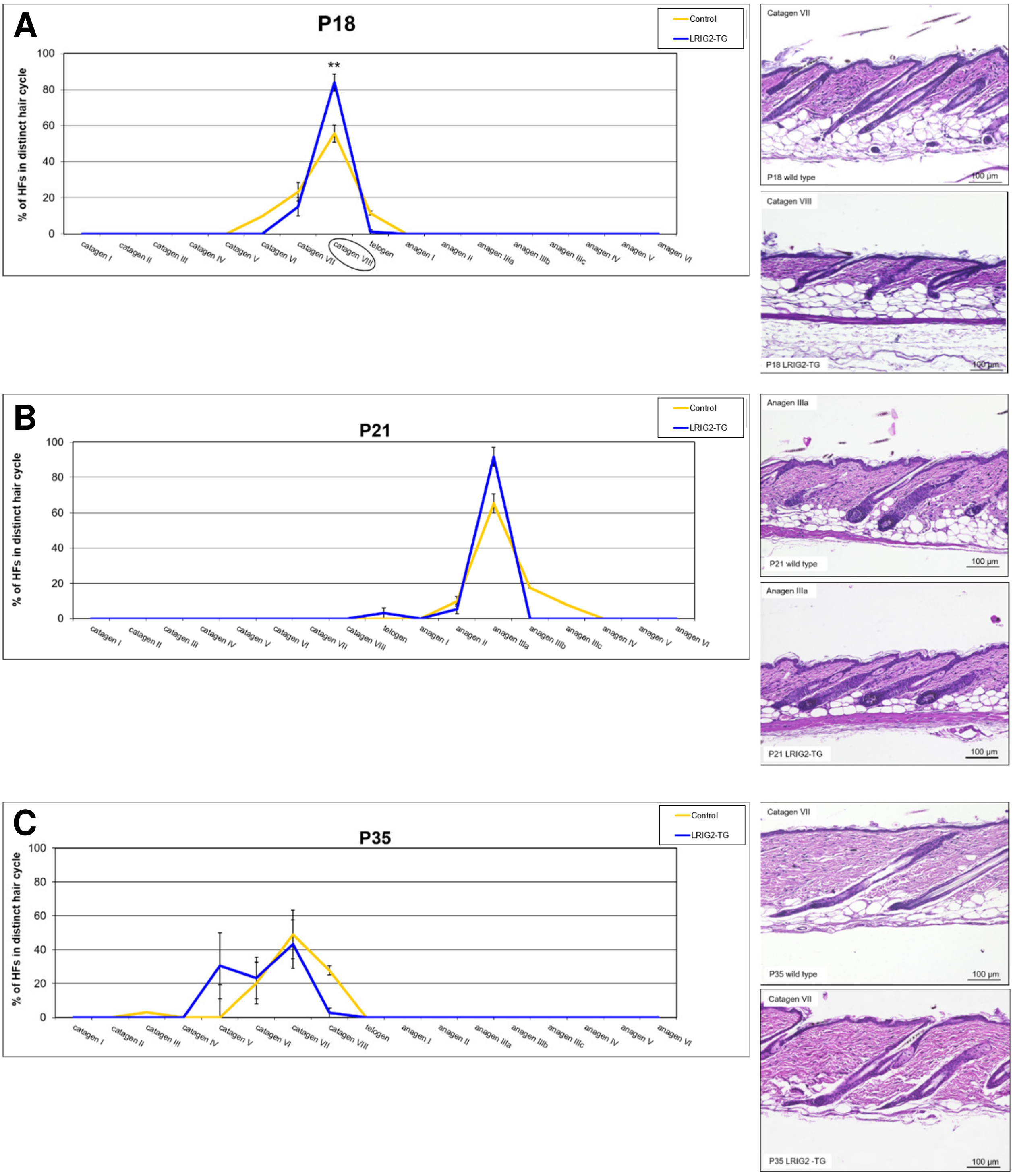

Figure S4. The hair follicle cycle in LRIG2-TG mice. Hair follicles (HFs) of controls and LRIG2-TG mice on days post partum (P) 18, 21 and 35 were evaluated according to the HF cycling phases as described elsewhere [1]. The total number (%) of HFs in distinct HF cycle phases was counted in LRIG2-TG and wild type mice; mean ± SEM, using Mann-Whitney *U*-test, $^{**}P \leq 0.01$, n= 3-5, representative H&E stainings of a LRIG2-TG and a control mouse are shown for each P. Scale bars represent 100µm. **(A)** LRIG2-TG mice on P18 show significantly more HFs in the late catagen phase VIII compared to controls. **(B)** There are no differences in HF cycle phases on P21 between control and LRIG2-TG mice. **(C)** No remarkable differences in the HF cycle phases on P35 can be found between control and LRIG2-TG mice.

([1] Muller-Rover S, Handjiski B, Van D, V, Eichmuller S, Foitzik K, McKay IA, Stenn KS, Paus R: **A comprehensive guide for the accurate classification of murine hair follicles in distinct hair cycle stages.** *J Invest Dermatol* 2001, **117:**3-15.)

Hoesl et al.
Fig. S4
