# Supplementary material for: The transmembrane protein LRIG2 increases tumor progression in skin carcinogenesis
Source: Mol Oncol. 2019 Oct 21;13(11):2476–92. doi: 10.1002/1878-0261.12579 (PMC6822252; doi:10.1002/1878-0261.12579)
Supplement: Supplementary file 5 — Fig. S5. Mass spectrometry data. [file MOL2-13-2476-s005.pdf]

A

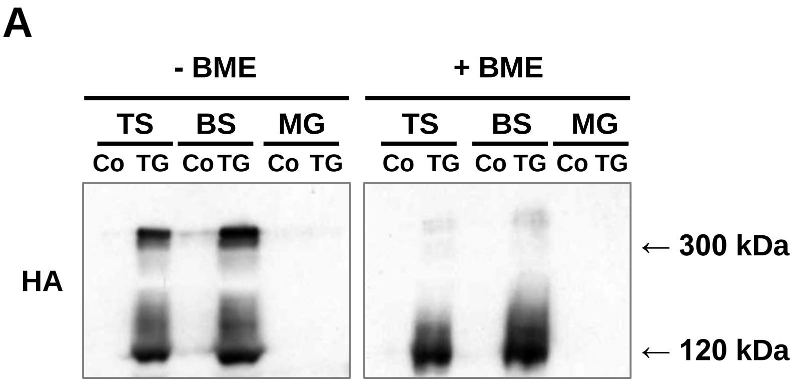

B

	Protein	Molecular Weight		Protein	Molecular Weight
1	KRT71	57 kDa	11	ATP1B3	56 kDa
2	KRT31	47 kDa	12	HSPA1A	70 kDa
3	KRT27	49 kDa	13	ACTN3	103 kDa
4	LAMB1	197 kDa	14	ACTN2	104 kDa
5	KRT83	55 kDa	15	UBA1	118 kDa
6	KRT75	60 kDa	16	LRIG2	118 kDa
**7**	**THSB1**	**130 kDa**	17	LAMA2	343 kDa
8	TPM2	33 kDa	18	MDH2	36 kDa
9	PYGM	97 kDa	19	ANXA2	39 kDa
10	SLC4A1	103 kDa	20	PADI2	76 kDa

C

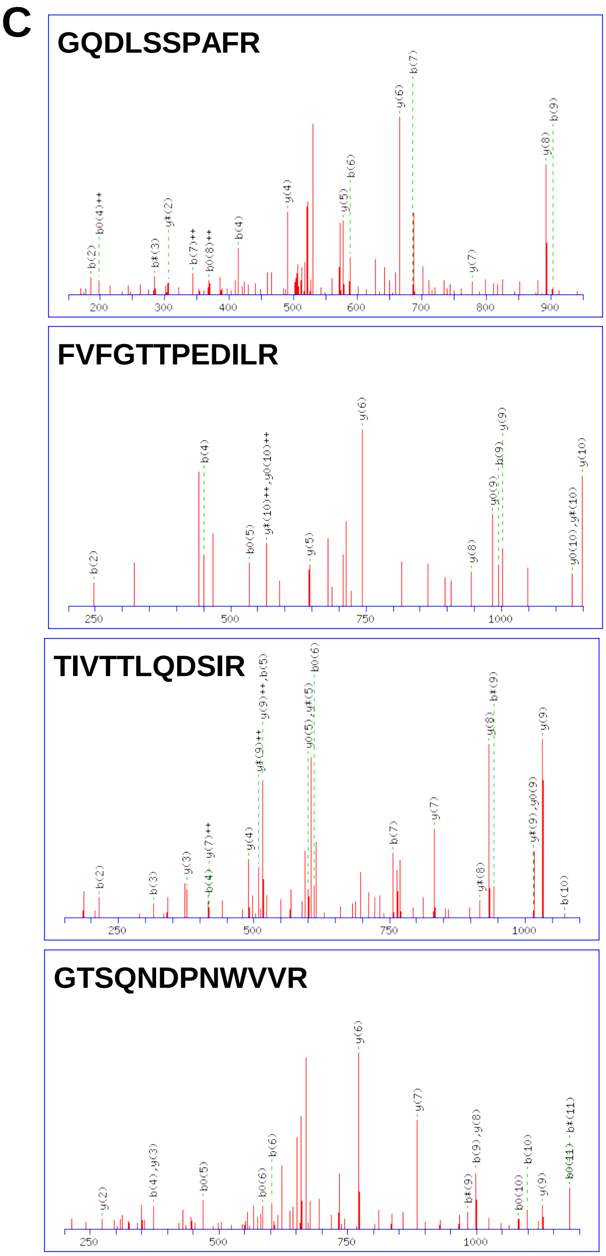

D

Sequence	Prob	Mascot Ion score	Observed	Actual Mass	Charge	Delta Da	Delta PPM	Start - End
(K)GQDLSSPAFR(I)	100%	26.29	539.2702	1,076.53	2	0.0006781	0.6293	51 - 60
(R)FVFGTTPEDILR(N)	100%	27.49	697.87	1,393.73	2	0.001078	0.7729	217 - 228
(R)FVFGTTPEDILR(N)	100%	28.29	697.8701	1,393.73	2	0.001278	0.9163	217 - 228
(R)TIVTTLQDSIR(K)	100%	37.36	623.8544	1,245.69	2	0.001178	0.9449	289 - 299
(R)TIVTTLQDSIR(K)	100%	25.02	623.8541	1,245.69	2	0.0005781	0.4637	289 - 299
(K)GTSQNDPNWVVR(H)	100%	30.49	686.8333	1,371.65	2	-0.001122	-0.8173	969 - 980

Figure S5. (A) Western blot analysis reveals an additional signal for LRIG2 at 300 kDa under non-reducing conditions. Co: control, TG: transgen, TS: tail skin, BS: back skin, MG: mammary gland, +/-BME: with/without beta-Mercaptoethanol. **(B)** List of top 20 proteins which were exclusively identified around 300 kDa in LRIG2-TG samples and not in controls sorted by total spectral counts of the non-reduced LRIG2-TG protein fraction. **(C)** Spectra of four individual TSP1 peptides. **(D)** Identified TSP1 peptides with probability and MASCOT scores.

Hoesl et al.
Fig. S5
