# Supplementary material for: The transmembrane protein LRIG2 increases tumor progression in skin carcinogenesis
Source: Mol Oncol. 2019 Oct 21;13(11):2476–92. doi: 10.1002/1878-0261.12579 (PMC6822252; doi:10.1002/1878-0261.12579)
Supplement: Supplementary file 6 — Table S1. Antibodies employed for Western blots analysis, immunoprecipitation, immunohistochemistry, and immunofluorescence. [file MOL2-13-2476-s006.pdf]

Table S1. Antibodies employed for Western blots analysis, immunoprecipitation, immunohistochemistry, and immunofluorescence.

Antigen	Antibody	App.	Host	Dilution
LRIG2	GeneTex, Alton Pkwy Irvine, CA, USA, #37384	WB	rabbit	1:500
LRIG2	LSBio, Seattle, WA, USA, #C165865	IHC	rabbit	1:100
LRIG2	Abcam, Cambridge, UK, #121472	WB	rabbit	1:500
HA-tag	Cell Signaling, Boston, MA, USA, #3724	WB/IF	rabbit	1:1000/1:200
HA-tag	Abcam, Cambridge, UK, #9110	WB/IP	rabbit	1:5000
LRIG1	R&D Systems, Minneapolis, MN, USA, #AF3688	WB	goat	1:2500
LRIG3	R&D Systems, Minneapolis, MN, USA, #MAB3495	WB	mouse	1:1000
p-EGFR (Tyr 1068)	Cell Signaling, Boston, MA, USA, #3777	WB	rabbit	1:1000
p-EGFR (Tyr 1086)	Cell Signaling, Boston, MA, USA, #2220	WB	rabbit	1:1000
p-EGFR (Tyr 1173)	Santa Cruz, Heidelberg, Germany, #12351,	WB	rabbit	1:500
EGFR	Santa Cruz, Heidelberg, Germany, #03,	WB	rabbit	1:500
EGFR	R&D Systems, Minneapolis, MN, USA, #AF1280	IF	goat	1:200
p-ERBB2 (Tyr 877)	Cell Signaling, Boston, MA, USA, #2241	WB	rabbit	1:1000
p-ERBB2 (Tyr 1221)	Cell Signaling, Boston, MA, USA, #2243	WB	rabbit	1:1000
ERBB2	Cell Signaling, Boston, MA, USA, #4290	WB	rabbit	1:1000
ERBB2	R&D Systems, Minneapolis, MN, USA, #AF5176	IF	sheep	1:200
p-ERBB3 (Tyr 1289)	Cell Signaling, Boston, MA, USA, #4791	WB	rabbit	1:1000
ERBB3	Cell Signaling, Boston, MA, USA, #12708	WB	rabbit	1:1000
ERBB3	R&D Systems, Minneapolis, MN, USA, #AF4518	IF	sheep	1:1000
p-ERBB4 (Tyr 1258)	Abcam, Cambridge, UK, #76132	WB	rabbit	1:1000
ERBB4	Santa Cruz, Heidelberg, Germany, #283	WB	rabbit	1:500
ERBB4	Santa Cruz, Heidelberg, Germany, #8050	WB/IF	mouse	1:500/1:50
THBS1	Cell Signaling, Boston, MA, USA, #37879	WB	rabbit	1:1000
p-MAPK1/2 (Thr202/Tyr204)	Cell Signaling, Boston, MA, USA, #4370	WB	rabbit	1:1000
MAPK1/2	Cell Signaling, Boston, MA, USA, #9102	WB	rabbit	1:1000
p-AKT (Ser 473)	Cell Signaling, Boston, MA, USA, #4060	WB	rabbit	1:2000

AKT	Cell Signaling, Boston, MA, USA, #4691	WB	rabbit	1:1000
p-PTEN (Ser380/Thr382/383)	Cell Signaling, Boston, MA, USA, #9554	WB	rabbit	1:1000
PTEN	Cell Signaling, Boston, MA, USA, #9552	WB	rabbit	1:1000
PCNA	Cell Signaling, Boston, MA, USA, #13110	WB	rabbit	1:1000
CASP3	Cell Signaling, Boston, MA, USA, #9662	WB	rabbit	1:1000
IL1A	R&D Systems, Minneapolis, MN, USA, #AF400NA	WB	goat	1:2000
IL6	Cell Signaling, Boston, MA, USA, #12912	WB	rabbit	1:1000
MKI67	Dianova, Hamburg, Germany, #M7249	IHC	Rat	1:200
KRT5	BioLegend, San Diego, CA, USA, #905501	IF	rabbit	1:800
KRT6	BioLegend, San Diego, CA, USA, #905701	IF	rabbit	1:800
KRT8	BioLegend, San Diego, CA, USA, #904801	IF	mouse	1:200
KRT10	BioLegend, San Diego, CA, USA, #905701	IF	rabbit	1:800
LOR	BioLegend, San Diego, CA, USA, #905101	IF	rabbit	1:800
CDH1	R&D Systems, Minneapolis, MN, USA, #AF748	IF	goat	1:200
VIM	Cell Signaling, Boston, MA, USA, #5741	IF	rabbit	1:200
TUBA1A	Cell Signaling, Boston, MA, USA, #2125	WB	rabbit	1:1000
GAPDH	Cell Signaling, Boston, MA, USA, #2118	WB	rabbit	1:5000
BrdU	AbDSeroTec, Puchheim, Germany, #OBT0030	IHC	rat	1:100
donkey α rabbit	Jackson ImmunoResearch, Ely, UK, #711-546-152	IF	donkey	1:1000
donkey α goat	Jackson ImmunoResearch, Ely, UK, #705-545-147	IF	donkey	1:1000
donkey α sheep	Jackson ImmunoResearch, Ely, UK, #713-586-147	IF	donkey	1:1000
donkey α mouse	Jackson ImmunoResearch, Ely, UK, #715-585-140	IF	donkey	1:1000
mouse α rat	Jackson ImmunoResearch, Ely, UK, #212-066-168	IHC	mouse	1:100
rabbit α rat	AbDSeroTec, Puchheim, Germany, #STAR21B	ICH	rabbit	1:100
rabbit α mouse	Cell Signaling, Boston, MA, USA, #7076	WB	rabbit	1:2500
goat α rabbit	Cell Signaling, Boston, MA, USA, #7074	WB	goat	1:2500
goat α rat	Cell Signaling, Boston, MA, USA, #7077	WB	Goat	1:2500
donkey α goat	R&D Systems, Minneapolis, MN, USA, #HAF109	WB	donkey	1:2500
